# In silico imaging clinical trials: cheaper, faster, better, safer, and more scalable

**DOI:** 10.1186/s13063-020-05002-w

**Published:** 2021-01-19

**Authors:** Aldo Badano

**Affiliations:** Division of Imaging, Diagnostics, and Software Reliability, Office of Science and Engineering Laboratories, Center for Devices and Radiological Health, US Food and Drug Administration, Silver Spring, MD USA

**Keywords:** In silico trials, Computational modeling, Regulatory evaluation

## Abstract

Imaging clinical trials can be burdensome and often delay patient access to novel, high-quality medical devices. Tools for in silico imaging trials have significantly improved in sophistication and availability. Here, I describe some of the principal advantages of in silico imaging trials and enumerate five lessons learned during the design and execution of the first all-in silico virtual imaging clinical trial for regulatory evaluation (the VICTRE study).

## In silico imaging

Imaging clinical trials aim at answering specific scientific questions regarding the value of imaging technologies and procedures for detecting, diagnosing, guiding, or monitoring the treatment of disease. Imaging clinical trials can be burdensome for industry and regulators, often delaying patient access to novel, high-quality medical devices. The evaluation of novel imaging technologies typically requires a substantial clinical study to demonstrate benefits compared to the standard of care. While computational models are sometimes used in the regulatory evaluation of medical devices, their use in support of imaging products has been rare. However, tools for in silico imaging trials have significantly improved in sophistication and availability since the late 1980s and particularly since 2000, with refined and efficient freely available tools increasingly being used in research and development.

The term “in silico imaging” has been defined as the computer simulation of an entire imaging system including source, object, detection, and image interpretation components used for research, development, optimization, technology assessment, and regulatory evaluation of new technology to complement bench testing [1]. This broad definition expands the uses of the term beyond the more conventional applications in research and development of new imaging technology into areas where computer simulation has not yet been applied at any significant level. For instance, simulation tools can be used by industry and regulators to better understand modifications to existing devices and to predict the performance of new technology.

While many investigations on the use of in silico imaging to study the performance of radiation imaging systems (see, for instance, [2]) have been reported, no all-in silico clinical imaging trial has been reported until recently [3].

The VICTRE study consisted of an in silico replication of a comparative human trial demonstrating the potential of this emerging approach to encourage widespread use of in silico trials for regulatory evaluation. The VICTRE project’s primary goal was to demonstrate the current maturity of the in silico tools and provide evidence that similar regulatory decisions could be made based on in silico evidence at a fraction of the cost of a clinical trial, the latter involving imaging of hundreds of patients collected in several clinical sites and across many years. Although VICTRE’s conclusions were promising, barriers to the widespread adoption of in silico techniques for clinical trials remain.

This article describes some of the principal lessons learned during the design and execution of the VICTRE study, in an effort to encourage and provide guidance to others contemplating performing in silico imaging clinical trials. It is presented from the perspective of the researcher in charge of scoping, designing, and conducting the in silico trial and begins with a review of key aspects of the VICTRE study, followed by a description of the top five lessons learned accompanied by examples drawn from the VICTRE project.

## The VICTRE study

VICTRE (Virtual Imaging Clinical Trial for Regulatory Evaluation) was an in silico clinical imaging trial evaluating digital breast tomosynthesis (DBT) as a replacement for digital mammography (DM). The results of the simulated trial were compared to those of a previously conducted human clinical trial [4] that double-exposed more than 400 women to both modalities and had images interpreted by radiologists.

In VICTRE, images obtained with in silico versions of DM and DBT systems via detailed Monte Carlo x-ray transport were interpreted by a computational reader using a performance task in which the target shape and location were known a priori and shape did not vary from patient to patient or case to case (e.g., signal-known-exactly task).

A total of 2986 synthetic patients with breast sizes and radiographic densities representative of a screening population and compressed thicknesses from 3.5 to 6 cm were generated using an analytic approach in which anatomical structures are randomly created within a predefined volume and compressed in the craniocaudal orientation. Digital patients were imaged using in silico DM and DBT systems. A cancer-present cohort contained digitally inserted microcalcification clusters or spiculated masses.

**Fig. 1:**
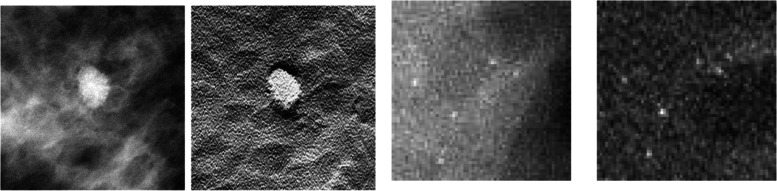
Example image regions containing a lesion. (from left to right) A spiculated mass shown embedded in the same location of a digital breast model seen in a DM and DBT image. The absorption of the lesion has been increased to ensure conspicuity for this illustration. A microcalcification cluster embedded in the same location of a digital breast model seen in a DM and DBT image. The DBT images correspond to a slice of the three-dimensional volume obtained from reconstructing the multiple angular projections

Figure 1 illustrates the differences in the presentation of the lesions in both modalities. The mass appears more demarcated in the DBT image which represents a single slice of the three-dimensional image DBT reconstruction. Both the lesions and the anatomical backgrounds are represented differently in both modalities, contributing to the difference in diagnostic performance.

The in silico trial end point was the difference in area under the receiver operating characteristic curve between modalities for lesion detection. The trial was sized for a standard error of the mean (SE) of 0.01 in the change in area under the curve (AUC), half the uncertainty in the comparative clinical trial. The results of the in silico trial were analyzed and reported for 31,055 DM and 27,960 DBT images obtained from 2986 patient images with the following Breast Imaging Reporting and Data System densities: 286 (9.6%) extremely dense, 1200 (40.2%) heterogeneously dense, 1200 (40.2%) scattered fibroglandular densities, and 300 (10.0%) almost entirely fat.

The mean (SE) change in AUC for the VICTRE study was 0.0587 (0.0062) in favor of DBT, consistent with the comparative trial. The change in AUC was also consistent with the comparative trial results in that it was larger for masses (mean [SE], 0.090 [0.008]) than for calcifications (mean [SE], 0.027 [0.004]). A detailed statistical analysis of the VICTRE trial results can be found in Ref. [5].

The consistency of the results of the in silico and comparator trials is indicative of the general soundness of the model assumptions. However, one has to be careful not to extrapolate the models to other conditions or problems or comparisons where they might fail. By any means, this is a fairly young field of research with much yet to be understood about the robustness of the in silico tools.

## Limitations

In silico imaging clinical trials have limitations that are worth noting. Any clinical trial accruing cases from a patient population (asymptomatic if the trial is for screening) results in a wide range of patient characteristics. In the case of breast imaging trials, the age, ethnicity, breast size, and breast density of patients enrolled in a study approach a target distribution defined a priori within the design phase of the trial. This variability applies to patients with and without abnormalities as well as to the abnormalities themselves. Because of this variability, lesions seen in the trial range widely in size, attenuation properties, and other morphological features as seen in the x-ray image. This variability was not considered in the modeling of the VICTRE patient population. The trial consisted of four breast shapes (sizes) each with a corresponding average breast density. With sufficient data, a statistical description of the relationship between breast morphology and density (perhaps extracted from a large database of images) could be implemented to produce a population of patients that most closely mirrors the characteristics of actual patients. The VICTRE patients exhibiting abnormalities had only one of two lesions (a spiculated mass or a microcalcification cluster) each with a unique size.

In addition, VICTRE lesions were inserted after the breast models were physically compressed using finite-element methods, therefore ignoring the distortions introduced by the presence of lesions in the surrounding normal breast structures. This limitation of the VICTRE models could be easily surmounted with knowledge of the physical properties of the lesions, and would nevertheless have a similar effect on both imaging modalities (DM and DBT).

Even though patients’ breasts during the DM and DBT examinations were physically compressed, patient motion introduces blur. Because the scan time of DBT is many times longer than the scan time for DM, motion blur could affect DBT more than DM. VICTRE models did not consider patient motion. Motion during x-ray imaging procedures for the breast is a topic of current interest [6], and once models become available, they could be easily incorporated in future imaging simulation efforts.

Finally, a major area in need of refinement in the VICTRE study methodology was the modeling of the medical decision-making with respect to each image. In the human trial, radiologists interpreted a full case by reviewing mediolateral-oblique and craniocaudal views of both breasts, and determined the likelihood of the patient having a cancerous lesion. The radiologists’ task involved searching over the entire image (DM) or entire image set (DBT) for suspicious regions and determining the probability of malignancy.

In the in silico VICTRE study, only the algorithmic decision-maker determined the presence of a lesion in a known location by inspecting regions (DM) or volumes (DBT) of interest. Searching was not considered in the VICTRE tasks for either DM or DBT images. While it is known that searching patterns for DM and DBT ought to be different, it is not known how search affects the performance of the modalities relative to a comparison based on lesion location known exactly. Search remains a topic of interest in the modeling community, especially in 3D image stacks.

In the end, the findings of the VICTRE study would have resulted in a similar regulatory decision to that reached by the Food and Drug Administration (FDA) using the comparative human trial. While the results of the VICTRE study are not immediately generalizable to other imaging devices and further investigations are needed to provide necessary validation of the in silico clinical imaging trial approach, progress is promising in this area.

To encourage other such efforts, I first provide a rationale for the advantages of in silico trial approaches and then describe the most important lessons that I compiled as the VICTRE study went from the initial design phases to the execution and analysis phases.

## Advantages (and disadvantages)

Currently, in silico imaging clinical trials have strengths and weaknesses much like trials employing human patients and physicians. Table 1 summarizes the main differences. Chiefly, the in silico trials are always approximations of the physical world and that might constitute a limitation, while real-world trials employ the devices as constructed. The modeling approximations affect the aggregate performance of the device and its variability but, more subtly, could ignore aleatory phenomena that might exist in the physical world and have been omitted in the simulated space.

**Table 1:** Strengths and weaknesses of human and in silico imaging clinical trials

Human trial	In silico trial
+ Real-world device usage	−Approximation
− Limited samples	+ Unlimited samples
− Large variability	+ Adjustable variability
− Narrow scope	+ Broader scope
− Truth uncertain	+ Truth known exactly
− Risk to patients	+ No patient risk
− Burdensome	+ Less burdensome

Unlike in silico trials, most human trials are limited in the number of samples that can be incorporated (or in the accrual rate) into the study by the low prevalence of the diseased conditions and rely on enrichment strategies.

In addition, image interpretation variability is significant and must be considered in the design of the trial, while in silico image interpretation can be designed at any variability level.

Another aspect of human trials is that because they are expensive and lengthy, they are typically designed with a narrow scope, or in other words, to answer a specific question regarding the performance of the devices. In silico imaging trials, on the other hand, can be designed to answer a rich variety of relevant questions by providing sufficient statistical power to test different hypothesis with a relatively small increase in resources associated with evaluating more models once the computational machinery is developed and implemented.

Even within the narrow scope of the trials, it is often the case that establishing the truth state of samples is subject to some degree of uncertainty while in silico trials can be designed with truth state exactly known.

Finally, human trials involve procedures on patients with inevitable additional risks including that of exposure to ionizing radiation. Perhaps the most significant difference is the larger resource expenditures required to conduct human trials.

Conducting trials with computational methods is challenging, primarily because of the incipience of the field, as well as the community’s skepticism. The latter is laudable, since the implications are significant and affect us all. However, as the field of computational trials becomes progressively more and more established, the reasons for interest in in silico imaging trials will become clear: cheaper, faster, better, safer, and more scalable than human trials. Here, I provide the rationale behind each on of these advantages relying primarily on examples from the VICTRE study.

### Cheaper

A chief advantage of in silico clinical trials is the substantial cost savings. Resource savings depend on the device modeled and imaging system characteristics, on the relevant disease prevalence, and on the availability and accessibility of the targeted population. Our estimates indicate that VICTRE study required only one third of the resources required to design and complete the comparative trial. Using approximately three full-time researchers, the VICTRE study’s expenditure in scientist-hours was comparable to that of the comparative trial. The comparative trial took approximately 4 years to complete, versus 1.75 years for the design, implementation, and execution of the VICTRE trial.

Operational costs including patient recruitment were similar to the computational costs in VICTRE. These estimates disregard savings including the costs associated with the risk from double-exposing hundreds of women to ionizing radiation. The estimates also ignore costs associated with institutional review board approvals and clinical facilities. Not only are these estimates conservative, but they also are likely to increase in the future as computing resources become less expensive.

### Faster

In calculating the time required to perform a clinical imaging trial, we need to consider the time allocated to recruiting patients, the time performing procedures and ensuring patients return to the clinic for follow-up or for additional imaging procedures. While imaging procedures typically take only a few hours, recruiting patients can take months. A recent study showed that patient recruitment is a key determinant of success for clinical trials [7]. The report also showed that many trials are delayed, or terminated early because of low accrual and high dropout rates. In addition, interpretation of the images by experienced radiologists must be scheduled and adds additionally delays the completion of the trial. During an in silico imaging clinical trial, once the characteristics of the patient population are determined in the design phases of the study, 24 h is typically sufficient for patient model generation, model preparation for imaging (including physical compression), imaging procedures, and imaging data processing (including 3D image reconstruction) and analysis (image interpretation) [8]. With the advent of massively parallel computer platforms with thousands of processing cores, today it is possible to execute a complete imaging clinical trial with thousands of patients in a day.

### Better

In silico imaging trials will soon become more useful than the limited and expensive human trials. For instance, it is possible to design an in silico imaging trial to study the performance of an imaging device in a subpopulation which might be difficult to include in human trials due, for example, to an extremely low prevalence of the condition to be imaged, or due to ethical concerns over the radiation exposure of a patient group (e.g., neonates and infants).

In silico methods can be designed for understanding how a novel drug or device affects specific digital patients with unique characteristics. For example, in silico breast imaging studies can provide insights into the improvements of performance for novel imaging techniques for a given virtual patient taking into account the anatomical structures and characteristics of the patient’s breast model. In other words, in addition to understanding the effect of the technology on a distribution of patients (e.g., a screening population), the in silico study could reveal improvements on a per patient basis allowing for the identification of patient characteristics that significantly influence the differential performance among technologies.

Moreover, as is the case in other industries, investigations can be conducted using computational models of devices that have not yet been built in the physical world, enabling a more efficient research and development cycle.

### Safer

Many of the errors seen in human trials including data errors and selection bias can be minimized with a carefully conducted in silico trial. The in silico methods should be fully reproducible both in the sense of recreating the trial and in the sense of controlling the pseudo-random generation of numbers needed in different stages of the modeling chain including components relying on Monte Carlo approaches or those based on random realizations of procedural models. Under this assumption, errors are virtually eliminated. In computational trials, patients do not drop out and are always available for additional testing. In silico trials are also safer in that there are no additional risks due to exposure, or in some cases, double-exposure of patients to ionizing radiation for imaging procedures. The patients that were recruited in the comparative trial for the VICTRE study were exposed to ionizing radiation at twice the typical levels for a breast x-ray exam to be able to correlate each patient imaged under both DM and DBT modalities. As a result, applications of in silico imaging in pediatric and neonatal procedures which involve patient populations more sensitive to ionizing radiation are of interest.

### More scalable

Performing an in silico trial such as VICTRE enables investigators to consider, without significant delays or additional resources, follow-up studies to look at additional questions which might have been inspired by the initial trial. Moreover, additional arms of the same trial can be performed immediately. For instance, the same patient population could be studied for another modality without having to reexpose them using modalities for which data has already been acquired. In addition, more powerful subpopulation analyses can be performed easily by enriching the population segment of interest. This can be performed without limitations in silico, including some impossible (or at least far impractical) to study with human patients (e.g., effects of extremely rare breast sizes or extremely low prevalence type of tumors on the performance of the devices). Moreover, and of great relevance to regulatory evaluation efforts, further technological iterations can be tested with minimal additional expenditures.

## Five lessons

Several lessons from the VICTRE study are worth considering before attempting to migrate from clinical trials to a computational approach. In this context, it has proven useful to be humble, expansive, and comparative; to oversize it; and to be aggressive in modeling. This section provides commentary on these five lessons and illustrates their relevance to the VICTRE study.

### Be humble

In silico surrogates are not and will never be identical to reality; understanding this relieves us from pursuing the impossible. It has been reported that in silico trials could in some cases provide evidence that is not or cannot be found in traditional approaches [9]. In other words, in silico trials could be better (or worse), but never identical. For example, the computational breast models used in VICTRE did not contain pectoral muscles. This significant anatomical landmark, however, remained of little importance with respect to the comparative results of the in silico and human trials. It is obvious that predictions from models not always match data collected from the physical world. A continuous effort to make sure the models track with real-world data is necessary. When modeling results are inconsistent with real-world data, research efforts need to focus on the needed model improvements by identifying the contributions of each one of the modeling components to the observed discrepancy.

### Be expansive

Whenever possible, the modeler should cover a range of parameter values to determine if the results obtained in a specific realization of the trial are not supported by a slightly different set of input parameters. This is a common procedure in engineering design and is often referred to as a sensitivity study. An example of such study can be found in Ref. [10], in which the authors adopted a software verification and validation framework to evaluate the in silico trial pipeline and to test the simulation results using a set of sensitivity studies including focal spot size and radiation dose level.

### Be comparative

All available data including bench testing results in the open literature should be used to extend the comparison and challenge the models. While it is always difficult to exactly replicate in silico the conditions described in a bench test method, performing comparisons with test objects that are designed to characterize the imaging systems under controlled conditions may be possible. The comparisons performed as part of the VICTRE study are described in detail in the Supplementary online-only material for Ref. [3].

### Oversize it

The medical use of ionizing radiation is governed by the principle of ALARA (as low as reasonably achievable). The concept refers to the directive to use the least amount of ionizing radiation that ensures obtaining the information required from the study. In silico trials should also use an analogous ALARA (as large as reasonably achievable) principle. Reducing all uncertainties and extending the range of the simulation only improves the confidence on its findings. At the same time, larger studies pose demands on computational power and on the transferring, manipulation, and storage of large datasets. A reasonable, practical size and associated uncertainties should be pursued. The statistical design of the VICTRE study could have been improved by recruiting 30,000 in silico patients (instead of 3000), or employing 300 image interpretation algorithms (instead of 30), to obtain a smaller uncertainty in the trial outcome. However, the trial’s findings would have been essentially the same and the consistency with the results of the human comparative study would remain unchanged. Proceeding with a pilot study for sizing purposes and understanding the contributions to the overall uncertainty from the number of cases and readers [11] remains a preferred approach.

### Overmodel it

If in doubt, overmodel (i.e., the practice of devoting significant resources to the modeling and analysis of interesting phenomena of insignificant relevance to the outcome and findings of the in silico trial). At the same time, providing a clear justification for any simplification contained in the models is good practice. An example of this approach can be found in the VICTRE models for the detector physics which included detailed models of the each x-ray interaction in the a-Se layer (see supplementary material for Ref. [3]). One could argue that a simpler approach of convolving an image acquired at the entrance of (immediately before) the detector with a blur function based on experimental measurements would have been sufficient for capturing the fundamental degradation in spatial resolution and noise correlations in the imaging detector. However, that simpler approach would require an additional effort to prove appropriate.

## Outlook

In silico imaging clinical trials that attempt to compare two imaging modalities or two different implementations of an imaging technology have the additional benefit that the result is evaluated in comparative terms. This comparative scenario is beneficial since many of the assumptions in the modeling may affect both modalities in similar ways, minimizing the effect of modeling uncertainties. The commentary provided in this article does not necessarily apply to a study investigating a novel technology for which a comparator does not exist. In such cases, the requirements for validating the evidence generated in an in silico stand-alone trial would be significantly higher. In silico imaging trials might also suffer from model tweaking in favor of a particular technology and should always comply with well-accepted clinical trial techniques that prevent biased outcomes.

The utilization of computer simulations in healthcare is only going to increase with applications ranging from training decision-making algorithms [12] and medical practitioners to planning therapeutic interventions. In silico trials, broadly known as the set of computational tools that simulate relevant properties of patients, devices, and medical practitioners, will play a significant part in the evaluation of imaging devices for efficient and scientifically sound regulatory decisions. Incrementally but inexorably, in silico imaging approaches will become the most significant component of evidence for regulatory evaluations.

## Data Availability

See Ref. [3] for open-source data associated with VICTRE.
